# Effects of Reading Health and Appearance Exercise Magazine Articles on Perceptions of Attractiveness and Reasons for Exercise

**DOI:** 10.1371/journal.pone.0061894

**Published:** 2013-04-22

**Authors:** Melanie Pankratow, Tanya R. Berry, Tara-Leigh F. McHugh

**Affiliations:** Faculty of Physical Education and Recreation, University of Alberta, Edmonton, Alberta, Canada; University of Nottingham Malaysia Campus, Malaysia

## Abstract

**Objective:**

To examine the effects of reading exercise-related magazine articles (health, appearance, or control) and the moderating effects of exercise self-identity on reasons for exercise and perceptions of attractiveness, among women in first year university. An additional purpose was to use a thought listing technique, the results of which were examined for evidence of internalization of the exercise-related messages.

**Participants:**

Female students in their first year of studies between September 2010 and April 2011 (N = 173; mean age = 19.31 years, mean body mass index = 22.01).

**Methods:**

Participants read a health, appearance, or control article, listed thoughts, and completed questionnaires measuring reasons for exercising, physical self-perception, and exercise self-identity.

**Results:**

Participants in the health condition rated exercise for health significantly higher than control condition participants. Participants with high exercise self-identity rated attractiveness as a reason for exercising significantly higher than low exercise self-identity participants in both the health and appearance conditions. Participants with higher internalization scores (i.e., accepted societal norms of appearance) reported exercising for attractiveness reasons more so than participants with lower internalization scores.

**Conclusions:**

The good news is that health messages may be influential and result in wanting to exercise for health purposes. However, exercising for attractiveness was rated highly by participants with high exercise identity who read either the health or appearance articles. Health and appearance are not necessarily distinct concepts for female undergraduate students and the media may influence cited reasons for exercise.

## Introduction

Despite proven health benefits of being active [1], the majority of Canadian adults do not engage in the recommended 150 minutes of moderate to vigorous physical activity per week [2]. Physical activity behaviors are important to study in first-year college and university students because physical activity and exercise behaviors tend to decrease significantly during the transition from high school to the first year of university [3]. This decline, which is greatest in females [4], can lead to health detriments during an individual’s lifetime, as many habits and behaviors that are learned and practiced during the years spent at college tend to extend further into adulthood [5].

It has been shown that low motivation to engage in physical activity, time constraints, and inconvenience were the most cited reasons for college students not participating in physical activity [6]–[7]. Conversely, the social benefits of exercising with friends and physical appearance improvements were important reasons for engaging in physical activity in college students [6]. At the same time that university students cite exercising for appearance reasons, negative changes in first year college students’ body self-perceptions have been reported [8]. Decreased body self-perceptions were attributed to weight gain during this time (average of 3.81 lbs/1.73 kg per year), which may in turn be due to a decrease in physical activity in first year students. A survey of 38, 204 male and female college students (mean age of 20.3 years) showed that 72.3% exercised with the main goal to lose weight, although very few were classified as being overweight or obese using body mass index (BMI) standards [9]. Weight loss was particularly important to the female participants.

Weight loss is often studied within the context of body image, a multidimensional construct that is comprised of perceptual, affective, and cognitive components of body experience [10]. Generally, women are more likely than men to report inaccurate and negative body weight perceptions, and these negative perceptions can manifest into weight loss attempts, often using inappropriate methods to do so [9]. Researchers have also suggested that negative body image perception is related to exercising for appearance-related reasons rather than for one’s health and wellbeing. A survey of 450 undergraduate students showed that female exercisers were found to have more preoccupation with their weight than female non-exercisers [11].

Such findings may be partly explained through sociocultural theory. The main supposition of this theory when applied to media is that societal standards in North America stress thinness and other ideas of beauty that are different from how most women look [12]. These societal ideals are pervasive in mass media that target young women and may form expectations regarding appearance and influence body image [13]–[14]. Thompson and colleagues [12] also argue that the media perpetuates the idea that the cultural ideal of beauty can be achieved, and the body changed, through diet and exercise. Indeed, popular fitness magazines use words such as “sculpt” when touting exercise routines purportedly designed to “transform one’s butt, abs, and thighs”. Thus, such magazines may establish norms of how women should look and give arguably unsubstantiated advice on how to achieve this look through exercise routines. A small amount of research has examined the effects of appearance related exercise messages on consumers. For example, researchers have found that exercise advertising featuring models that match societal ideals of beauty result in higher levels of body anxiety than advertisements that only included products [15]. These authors contend that the type of advertising seen in popular fitness magazines such as *Shape* can influence body image, but there is limited research examining this issue. There is also evidence some people implicitly believe that exercise will improve one’s appearance, even if they do not explicitly report such beliefs [16]. Although media representations of beauty can result in poor body image, Thompson and colleagues also point out that acceptance of these ideals is not universal and it is possible to reject them. It is those who are more accepting of societal standards of beauty (that is, they internalize them) who are more likely to develop body image issues.

Another model useful to this research is the elaboration likelihood model (ELM), which is commonly used in persuasion and advertising research [17]. The ELM takes into account the motivation to process an advertisement and the valence of cognitive processing (e.g., positive or negative), which is influenced by factors such as the attractiveness of the source and the number of arguments. Therefore, the ELM is a useful lens to reflect on how exercise advertising is processed and allows for consideration of differences in how health and appearance messages are presented. For example, appearance messages are more prevalent than health messages and rely on attractive models to “sell” exercise [16], [18]. A traditional thought listing technique used in advertising research [17], [19] may capture message processing and thus give information on how the advertising messages may have been internalized and the valence of processing (i.e., acceptance versus rejection of societal ideals of beauty).

Finally, it is important to consider the possibility that exercise identity may moderate these relationships. Identities are the self-perceptions a person has associated with a role (e.g., exerciser) and serve as standards for behavior [20]. Individuals are motivated to behave consistently with the meanings associated with an identity to which they relate [21]. Researchers have shown that exercise identity is related to exercise behavior [22] and to behavioral regulation related to exercise [23]. Those with high exercise self-identity may be less susceptible to appearance messages than those with low exercise self-identity because high self-identity is related to less controlled motives for exercise (e.g., health), and thus high exercise identity individuals may also report exercising more for health-related reasons after reading the health message [23]. This supposition is also made based on evidence that there is a strong relationship between beliefs in the health benefits of adults and exercise in young adults [24].

The purpose of this study was to examine the effects of reading exercise-related magazine articles (health, appearance, or control) on reasons for engaging in exercise, and body image perceptions, among young women in first year studies at university. The moderating effects of exercise self-identity were also examined and all analyses controlled for participant BMI. It was hypothesized that participants who read the appearance-related article would report appearance as an important reason to exercise [9], and negative perceptions of body attractiveness [13]–[14] in comparison to the other conditions. This hypothesis is based on sociocultural theory. It was also hypothesized that participants in the health condition would report health as an important reason to exercise because related research has shown that health messages result in more positive exercise-related attitudes and intentions to be active [16]. However, it was also hypothesized that these relationships would be moderated by exercise self-identity. Although there is little research directly assessing the relationship between exercise self-identity and body image, it is possible that higher self-identity participants will report more positive body image because of the strong relationship between exercise identity and behavior [22] and research showing that exercisers have more positive body image than non-exercisers [25]. Participants high in exercise self-identity in the health condition were hypothesized to report higher scores on exercise for health reasons because people with high exercise self-identity have been shown to have more autonomous reasons for exercise [23].

We also used a thought listing technique, in which participants wrote down thoughts they had while reading a message, to capture message processing [17], [19]. These data were examined to determine if participants showed evidence of internalization of the exercise-related messages. It was hypothesized that those who internalized the message would have worse body image, as predicted by sociocultural theory [12]. It was also hypothesized that those who internalized the message would rate appearance as a reason to exercise more so than those who reject the message. These data further provided context for the quantitative findings and also served as a manipulation check.

## Method

### Participants

Participants were first-year, undergraduate females (N = 188, mean age = 19.6 (SD = 2.87) years) from a Canadian university. This sample size is similar to related research [15]–[16]. Further, according to guidelines provided by Cohen [26], a minimum of 97 participants are needed for a regression with six predictor variables with alpha set to.05 and power = .80 and a medium effect size. Participants were recruited from introductory psychology classes who have the option of participating in research studies or completing assignments for credit. Students do not know the nature of the study when they sign up. Study information is provided when they report for testing. This study received ethics approval from the PER-ALES-NS ethics review board at the University of Alberta. All participants were university students and therefore had the capacity to give free and informed consent in the form of written informed consent and the ethics board approved that participants who were potentially minors (but university students) had the capacity to provide informed consent for themselves. All participants were given the opportunity to ask questions prior to participation.

### Materials

#### Magazine articles

Articles were selected from *Women’s Health* and *Shape* magazines because they are two of the top ten most read fitness magazines for women [27]. The first author selected articles believed to represent exercise for health or exercise for appearance. A pilot study was conducted with twenty female participants to confirm the primary messages in the articles were either health or appearance related. Each article was two pages long. The health-related magazine article was from *Women’s Health* magazine and was entitled *Can You Be Healthy at Any Size?* There was a large collage at the top of the page with images of a dozen celebrities of various body weights (e.g., Adele, Jennifer Hudson, Ellen Pompeo, Paris Hilton). The text of the article discussed whether it was healthier to be a bit heavier or to diet and be very thin, but the main message was that the key to being healthy is exercise and a healthy diet; size does not matter. The readability of the health article (calculated using Microsoft Office Word) was Flesch-Kincaid grade level 9.6 (i.e., the level at which a student in the ninth grade in the U.S. can understand). The appearance-related magazine article was from *Shape* magazine and was entitled *The Best-Butt Plan: Bikini Body Blitz.* This article included a photo of a fit, slender woman in a bikini and smaller photos of the woman demonstrating exercises on how to get a “bikini-body butt”. The text included directions on how to do the exercises and included a discussion of “fixes” for problems such as “saggy”, “bubble”, or “flat” butts. The readability of the appearance article was Flesch-Kincaid grade level 7.4. The control magazine article was from *People* magazine and entitled *Stepmom in the Spotlight*, which told the story of a missing boy in the United States, and had no content related to physical activity. The readability of the control article was Flesch-Kincaid grade level 8.6.

Pilot study participants were asked to rate each article for its health and appearance-related content with two questions on a 7-point scale (1 = strongly disagree to 7 = strongly agree): 1) “This article includes content related to exercising for my health,” and 2) “This article includes content related to exercising for my appearance.” Results for the health-related article showed high health content (mean = 6.1 [SD = .92]) and low appearance content (mean = 2.4 [SD = 1.08]), while the appearance-related article showed low health content (mean = 2.1 [SD = 1.38]) and high appearance content (mean = 6.7 [SD = 0.66]). The control article showed a mean of 1.1 [SD = 0.27] for both health and appearance-related content. Therefore, these articles represented health, appearance and neither (control) with very little overlap between conditions.

### Measures

#### Thought listing

Participants wrote down up to five thoughts they had while reading the articles in an open-ended format.

#### Demographics

Participants’ recorded their age (open ended) and ethnic background (“with which ethnic group do you most strongly identify” – open-ended responses). The participant’s ethnicities were placed into categories based on Statistic Canada’s 2006 Census of Canada Ethnic Origins [28]. Participants also self-reported their height and weight which was used to calculate body mass index (BMI). Participants were categorized according to the World Health Organization’s standard BMI measurements: underweight = <18.5, normal weight = 18.5–24.9, overweight = 25–29.9, obese = BMI of 30 or greater [29]. Participants also classified whether they perceived themselves as underweight, average weight, or overweight. This measure was compared to their actual body weight category calculated form their BMIs, to see if their self-perceived body size differed from their BMI according to WHO guidelines (albeit still self-reported).

#### Exercise self-Identity

Exercise self-identity was measured using a nine-item questionnaire rated on a 7-point Likert scale (1 = strongly disagree; 7 = strongly agree), which allowed participants to rate the extent to which they feel they are an exerciser or non-exerciser [22]. An example item is “others see me as someone who exercises regularly.” For the current study’s sample, Cronbach’s alpha = .93. A mean score of the nine items was created and the median split was used to create low and high exerciser self-identity groups.

#### Reasons for exercise

The 24 item Reasons for Exercise Inventory (REI), uses a 7-point Likert scale (1 = not at all important; 7 = extremely important) to assess the importance of various reasons for exercising [30]. In this study, the data from the health and attractiveness subscales were analyzed. Four statements assess health reasons for exercise such as “to improve my overall health,” and “to increase my resistance to illness and disease”. There are three attractiveness items such as “to improve my appearance” and “to be sexually desirable.” Mean scores were created from the items for each subscale. The Cronbach’s alpha for health = .75 and for attractiveness = .86.

#### Body image

Body image was measured using the body attractiveness subscale of the Physical Self-Perception Profile (PSPP). This questionnaire asks participants to choose between two opposing statements, either “really true for me” or “sort of true for me” [31]. Eight questions ask “What Am I Like”, and assess body attractiveness using statements such as “some people feel that they are often admired because their physique or figure is considered attractive” contrasted with “others rarely feel that they receive admiration for the way their body looks.” Three items were reverse scored so that a higher score indicates a more positive self-perception. Thus, marking “really true for me” on “others rarely feel they receive admiration for the way their body looks” was given a 1, “sort of true for me” a 2, a 3 for “sort of true for me” for the contrast statement of “often admired because their physique or figure is considered attractive”, and a 4 for “really true for me” for the contrast statement. Responses to the items were summed to create a body attractiveness score. Cronbach’s alpha for this study was.87.

### Procedure

Participation took place individually. Participants were given information about the study, and told that the study was testing thoughts and reactions to reading certain magazine articles. Participants were given the opportunity to ask questions, and were told that if they did not wish to continue the study at any time, an alternative activity was available to them. The alternative activity consisted of reading a journal article about physical activity and answering questions. None of the participants chose the alternative activity. Following informed consent, the participants were randomly assigned to one of the three conditions: health article, appearance article, or control article. After reading the article, participants recorded up to five thoughts they had while reading the article, followed by the questionnaires. To control for order effects, the questionnaires were given in a random order. After completing the questionnaires, the participants were debriefed and informed of the true nature of the experiment and had a chance to ask questions. The entire experiment took about 20 minutes to complete, with about five minutes spent reading the article.

## Results

Data from three of the participants were removed as outliers due to age (>31 years), and three with BMI >36 (univariate outlier criteria was +/−3.29 SD from the mean) [32], five participants did not complete all of the questions, two did not speak English well, one rushed through the experiment (i.e., clearly did not have time to read the article, did not record any thoughts, and recorded the same response for every questionnaire item), and one had been diagnosed with Fibromyalgia and Rheumatoid arthritis and was required to exercise (she volunteered this information to the research assistant). Thus, the data from 173 participants [mean age of 19.31 (SD = 1.80) years, range 17–28 years] were analyzed (60 in the health condition, 59 in the appearance condition, and 54 participants in the control condition). The most prevalent self-reported ethnic groups of the participants were North American (N = 39), East/Southeast Asian (N = 39), European (N = 34), and British Isles (N = 18). The remainder of the participants’ reported belonging to one or more of the following ethnic groups: Aboriginal, African, Arab, Latin/Central/South American, South Asian, and West Asian. BMI across the entire sample ranged from 16.24 to 35.66, with a mean BMI of 22.01 (SD = 3.36). Using BMI scores, 18 participants were underweight, 124 were average weight, 27 were overweight, and 4 were obese. There was no relationship between BMI and exercise self-identity, r = .07, *p = *.33. The mean score for exercise identity across all participants was 3.86 (SD = 1.48) with a range of 1–7 (skewness = −.102, kurtosis = −.842). Thus, exercise identity was evenly distributed and was on average lower than that in other studies with more active samples (e.g., Strachan & Brawley, 2008, reported a mean of 5.28 on this scale) [33]. Table 1 shows the demographic characteristics of the sample by experimental condition.

**Table 1:** Demographic characteristics of the sample by experimental condition.

		Health	Appearance	Control
Age (M [SD])		19.08 (1.51)	19.53 (1.88)	19.31 (1.99)
BMI (M [SD])		21.72 (2.76)	22.56 (3.84)	21.73 (3.39)
Identity (M [SD])		3.99 (1.56)	3.80 (1.55)	3.76 (1.32)
Self-reported weight (N [% within condition])	Underweight	1 (1.7%)	5 (8.5%)	4 (7.4%)
	Average weight	47 (78.3%)	37 (62.7%)	40 (74.1%)
	Overweight	12 (20.0%)	17 (28.8%)	10 (18.5%)

A series of one-way ANOVAs showed there were no differences between the article groups in age, BMI or exercise self-identity (all *p*>.29). A chi-square test showed no difference in ethnicity, χ^2^ (142) = 127.77, *p = *.80, or perceived weight, χ^2^ (4) = 5.22, *p = *.25, among the article groups. This indicates successful randomization.

### Differences between Article Conditions

The correlations between the dependent variables (DVs) were: REI-health and REI-attractiveness, r = .14, *p = *.07, REI-health and PSPP-body attractiveness r = −.015, *p = *.85, and REI-attractiveness and PSPP body attractiveness, r = −.31, *p*<.001.

One hierarchical linear regression model was conducted for each of the variables: REI- health, REI-attractiveness, and PSPP- body attractiveness. These outcome variables were regressed onto BMI in the first step, followed by experimental condition and exercise identity and their interactions in the second step. Following the recommendations of Aiken and West [34], experimental condition was dummy-coded so that the control condition was the comparison group, and exercise self-identity was mean-centered prior to calculating the interaction terms. These analyses tested the hypotheses that 1) participants who read the appearance-related article, compared to other conditions, would report appearance and body tone as important reasons to exercise and negative perceptions of body attractiveness and 2) that participants in the health condition would report health as an important reason to exercise. These models also tested the moderating effects of exercise self-identity.

**Figure 1:**
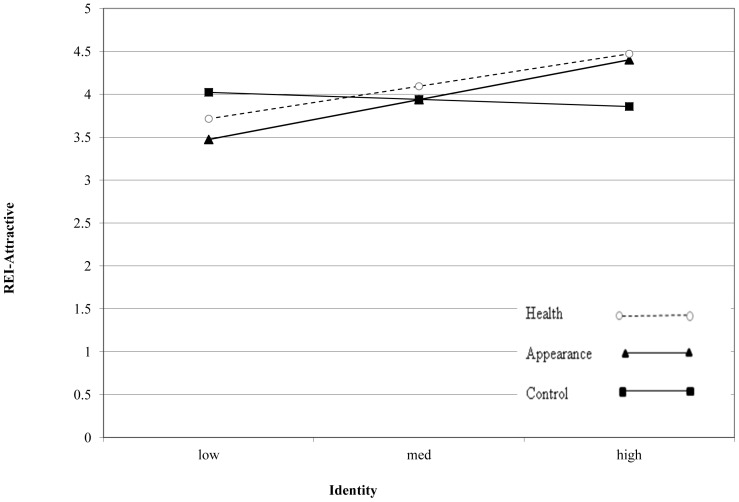
Simple slopes graph showing the relationship between REI-Attractiveness and identity by experimental condition.

Collinearity was not a problem in any of the models with all variance inflation factor (VIF) values <4.12 and tolerance values between 0.24 and 0.96. The models are summarized in Table 2. The only significant predictors of REI-Health were exercise identity and the dummy-coded variable that compared the health group to the control group. Participants with higher exercise identity rated exercise for health as higher, as did participants in the health condition compared to participants in the control condition. In the REI-Attractiveness model, the interaction between identity and the dummy-coded variable that compared the control group to the appearance group was significant. The interaction between identity and the dummy-coded variable comparing the control group compared to the appearance group predicted intentions, albeit not significantly (*p = *.06). Both these interactions were further explored using simple slopes analyses following the recommendations of Aiken and West [34] and the modgraph-I programme [35]. The results showed significant slopes for the participants in the health condition, t (169) = 2.44, *p = *.02, and the appearance condition, t (169) = 2.99, *p = *.003, but not for the control condition, t (169) =  −0.43, *p = *.67. Figure 1 shows a positive linear relationship between identity and REI-Attractiveness for those who read the health and appearance articles. The only significant predictor of PSPP-Attractiveness was BMI.

**Table 2:** Regression models predicting REI-Health, REI- Attractiveness, and PSPP-Attractiveness by experimental condition, exercise identity, and their interaction, also controlling for BMI.

			R^2^ Δ	B	Std.Error	β	*p*
REI - Health	Step 1	BMI	.003	−.014	.020	−.056	.467
	Step 2	BMI	.101*	−.021	.019	−.083	.269
		Identity		.199	.088	.337	.025
		D1		.454	.158	.249	.005
		D2		.210	.159	.115	.189
		D1 X identity		−.112	.112	−.118	.320
		D2 X identity		−.084	.113	−.087	.456
REI - Attractiveness	Step 1	BMI	.013	.044	.029	.114	.136
	Step 2	BMI	.087*	.036	.029	.095	.210
		Identity		−.056	.130	−.065	.665
		Condition D1		.153	.234	.057	.515
		Condition D2		−.003	.235	−.001	.990
		D1 X identity		.312	.166	.222	.062
		D2 X identity		.370	.167	.258	.028
PSPP - Attractiveness	Step 1	BMI	.191**	−.503	.079	−.437	<.001
	Step 2	BMI	.009	−.486	.082	.423	<.001
		Identity		−.298	.370	−.114	.421
		Condition D1		.317	.668	.039	.636
		Condition D2		.171	.673	.021	.800
		D1 X identity		.260	.473	.061	.584
		D2 X identity		−.009	.475	−.002	.984

* *p*<.01.

** *p*<.001.

*Note.* D1 = dummy variable comparing health to control; D2 = dummy variable comparing appearance to control; D1 X identity = interaction between D1 and identity; D2 X identity = interaction between D2 and identity; REI = Reasons for Exercise Inventory; PSPP = Physical Self-Perception Profile).

### Thought Listing Results

Four-hundred and fifty two thoughts were coded; 23 participants did not list any thoughts. There were 168 thoughts from the health article, 142 from the appearance article, and 142 from the control article. Thought listings were coded verbatim (i.e., thematic codes were assigned to the thoughts exactly as the participants wrote them). Following initial coding, one third were randomly selected using SPSS and coded by a different rater to determine inter-rater reliability. Themes were first generated with the aim of examining if the articles generated thoughts about health or appearance. In addition, codes were created according to the nature of processing: attitudes about health or appearance, motivation to exercise (or not), and reflections of affect (e.g., made the reader feel good, angry). Other codes were created as themes emerged. In this way, 13 codes were developed. Definitions and sample quotes are shown in Table 3. All thoughts from the control participants were rated as “other” and therefore not included in the table. The inter-rater reliability was adequate (kappa = .75). However, after discussion among the raters it was concluded there were some overlapping codes, and so some were collapsed and renamed and the data were recoded. The final kappa = .93. In the final coding there was 100% agreement between the raters on all codes with the exception of “fitness is key” in which the coders agreed on 24/27 of the statements with the second coder assigning the code “case for healthy fat” to three of the statements. The second coder also coded 1/12 of the “media targets women” statements as “case for healthy fat.” The only other discrepancy was two of the “not motivated to try changes” statements were coded as “media targets women” by the second coder.

**Table 3:** Final thought listing codes reflecting the processing of the messages, their definitions, example quotes and number of comments by health and appearance conditions.

Code	Definition	Example quote	Health	Appearance	Total
Case for healthy fat	Belief that thin does not equal healthy, thatfat people can be healthy and thinpeople unhealthy	Having a little extra fat may be healthy.	31	0	31
Case against healthy fat	Disbelief that being overweight or fat canin any way be healthy	I am shocked that some people are arguingthat extra fat is healthy.	9	0	9
Fitness is key; sizedoesn’t matter	Health has nothing to do with body weight,health depends on fitness	Fitness is more important than weight.	63	2	65
No solution to argumentof healthy fat	Both sides of the healthy/unhealthy fat arepresented with participant not sure whatto believe	I don’t think there will be a clear-cut solutionto the problem size vs health.	18	0	18
Motivated to try exercisesor changeappearance	Reading article motivated reader to be activeand to improve her appearance	Those seem like good exercises-Ishould try them!	0	38	38
Not motivated to tryexercises or changeappearance	Rejection of the message in the appearancearticle − reader does not want to“waste her time” doing the exercises or themessage is unrealistic	I wouldn’t put that muchwork into my bum.	0	20	20
Issues surroundingobese people	Comments only about obese people thatreflect the idea that by saying fat can be healthyit justifies people being “lazy”	Accepting “its ok to be fat” gives lazy,overweight people the idea that its ok notto exercise/eat healthy.	19	0	19
Media targets women	Messages in the media target women andmen are exempt from similar messages	pressure from peers or media everyday thatsay women must be “model” thin, otherwiseyou are not beautiful enough.	9	26	35
Readability (+)	Article was readable, easy to follow, enjoyable	Very easy to understand- clear defined steps	4	14	18
Readability (−)	Article was difficult to read or follow,not enjoyable	Instructions are daunting.	0	19	19
Summarizing Article	Participant just gave a summary with noadditional analysis or comments.	Workout 3–4 times a week, with at least oneday off between workouts.	3	10	13
Misc.	Doesn’t fit into any of the above codesor is irrelevant	Looks like an editorial out of a women’sfitness mag.	12	13	25

After the initial coding was established, themes were examined for evidence of internalization of the media message according to sociocultural theory. Because individuals differ in their awareness and acceptance of standards of beauty [12], the thought listings were given secondary codes regarding acceptance (i.e., internalization) of the appearance messages or rejection (lack of internalization) of the appearance message. Using the secondary codes and similar procedures to Berry et al. [16], a valence score was created for internalization (i.e., number of statements rejecting the standards of beauty presented in magazines subtracted from the number of acceptance statements). Thus, a positive valence score indicates internalization of the societal standard for appearance represented in the articles. Correlations between the valence score and REI- health, REI-attractiveness, and PSPP- body attractiveness were calculated. The thoughts that were coded as “motivated to try exercises or change appearance” characterized internalization of the societal ideal of beauty. An example internalization statement is “*tells you the type of improvement you’ll be doing on your butt, so that’s a plus.*” The thoughts that were coded as “not motivated to try exercise or change appearance” represented rejection of the societal ideal. An example statement is “*doesn’t account for the fact people having different body types is natural, not bad*.” In addition, thoughts coded as “media targets women” were also characterized as rejection of the ideal because they were critical of the media. Example statements include “*messages in the media are always about women, not men*” and “*should not focus on one part of the body, seems to only focus on what men like.*” The valence scores ranged from −3 [i.e., participants with this score made three statements made rejecting the societal ideal] to +3 [i.e., participants with this score made three statements made accepting the societal ideal].

Sixteen participants had internalization scores equal to or greater than 1 (all in the appearance condition) and 25 had scores of zero or lower (7 in the health condition and 18 in the appearance condition). The correlations between internalization and REI-attractiveness = .232, *p = *.008, between internalization and REI-health = .030, *p = *.732, and between internalization and PSPP-body attractiveness, =  −.159, *p = *.071. Participants with higher internalization scores, who were motivated by and accepting of the messages reported exercising for attractiveness reasons more so than those who rejected the ideal.

## Discussion

The purpose of this research was to examine whether reading exercise-related articles would be related to female university students’ reasons for exercise or their perceptions of their own body attractiveness. The hypotheses were partially supported. Participants in the health condition rated health as an important reason to exercise higher than participants in the control condition. It thus appears that the health article was successful in getting the participants to think about exercising for their health. This adds to a growing body of research showing the positive effect that exercise-related health messages can have on related cognitions [16], [18]. However, there was no main effect for exercising for reasons of attractiveness between article condition groups and the hypothesis that participants who read the appearance article would score higher on REI-attractiveness was not supported. This finding supports the contention of Thompson and colleagues [12] who argue that not everyone is susceptible to these magazines. Rather, in both the health and appearance conditions, participants with higher exercise self-identity rated exercising for attractiveness as more important than participants with low exercise self-identity. There was no significant difference in the control condition.

Participants high in exercise identity rated both health and appearance as important reasons to exercise. On the one hand, these findings support the work of Kennedy and Reis [11] who suggest that female exercisers are preoccupied with their physical appearance in relation to exercise. Others have similarly argued that individuals who exercise more often are more likely to imagine themselves exercising for their physical appearance to make a desired physical impression on others [36]. However, it was unexpected that the health article would also result in greater endorsement of exercising for attractiveness in those participants with high self-identity. It may be that the participants with exercise self-identity believe that being healthy and being fit is attractive. Identities involve the assimilation of role meanings (expectations associated with this role) into the self [21]. Health and appearance are strongly associated with exercise and thus an “exerciser” may consider both health and appearance to be very important. Qualitative research supports this notion – ideas of health, fitness, and appearance were interwoven among active college students [37]. Participants in that study discussed how the popular media influences standards of health and fitness and related them to appearance standards. Thus, high exercise identity participants in our study may have conflated health and attractiveness. If it is generally thought that exercise is a means to be more attractive, and one identifies as an exerciser, reading the health article may have elicited related thoughts which resulted in being healthy linked to attractiveness.

Indeed, the data generated in the thought listing indicate that many participants rejected the societal ideal and were not inclined to give in to the thin-ideal that is portrayed in the media, supporting Thompson and colleagues [12]. For instance, one participant wrote “I think the message of this article should be to embrace your body type and not worry about numbers.” Other research has also shown that even though young female participants recognized that there are certain ideals that young women are expected to achieve, they made conscious decisions not to necessarily succumb to such pressures [38]. Similarly, Paquette and Raine [39] found that body image perceptions in women fluctuate with experiences and interpretation of those experiences, and that the media’s influence is interpreted differently by every person. While many women in their study internalized the sociocultural norm of the thin-ideal, many also rejected the “ideal” and expressed acceptance in themselves and their bodies. In the current study, those who rejected this notion also had lower scores on exercising for attractiveness. Thirty-five specific comments were made regarding the pressure the media places on women to be thin. Participants who read the health article also made a number of comments regarding how fitness was much more important than size or body weight. Thus, the health article was able to elicit thoughts that countered ideas of the “thin-ideal” and that “fitness is more important than weight.” It is also possible that repetition of the appearance and exercise message results in counter-arguments, as posited by the ELM [17].

No difference was found between the conditions on perceptions of one’s attractiveness. This does not support the hypothesis and is in contrast to the findings of Tucci and Peters [40], who found that body image decreased and drive for thinness increased after a single exposure to a photo of a thin celebrity. We also did not find a relationship between exercise identity groups nor an interaction between article condition and self-identity on perceptions of one’s attractiveness. This again differs from other researchers. For example, it has been found that female exercisers had higher body image satisfaction than non-exercisers, as well as higher self-esteem, and lower social physique anxiety [25]. However, it may be that body weight is more strongly related to body image satisfaction than exercise identity, as BMI was a strong covariate in our analysis. The results of the current study suggest that messages about exercising for one’s appearance may not be as influential as previously thought and that health messages have a strong influence. It is also possible that due to the generally understood relationship between physical activity and health, a single exposure to an appearance-related article may not be enough to elicit a negative body image perception. Due to conflicting findings, further research is needed to determine the effects of appearance-related exercise articles on perceptions of one’s own attractiveness.

### Limitations

The strengths of this research include a randomized design with ecologically valid stimuli that are popular with the population from which our sample was drawn. However, there were a few limitations to this study. First, the participants may not have read the magazine articles thoroughly enough to be influenced. Although the participants were asked to list the main thoughts they had about the article immediately after reading it, some did not have any comments, and some thoughts were unrelated to article content. It is also possible that some participants may have just come from exercising, or may have been unable to exercise for an extended period of time. In either case, the participants’ answers to the questions may have been influenced by variables other than the magazine article. Self-reported height and weight is also a limitation. Future research should include objective measures of height, weight, and body composition so that accurate discrepancies between perceived weight and actual body size and composition can be determined. Further, the possibility of socially desirable responses is a potential limitation. It is also possible that participants in the health condition processed their article more than participants in the appearance condition as the health article was written in article format rather than the step-by-step instructions in the appearance article. However, in addition to the instructions, the appearance article also contained a half page discussion of “problem butts” and how to “fix” them. Finally, there is much unexplained variance in the findings. Future research should explore other possible factors such as prior exposure to the thin ideal and other aspects of body image beyond body attractiveness or reasons for exercise.

### Conclusions

These results are important for future researchers who wish to understand how media consumption may influence reasons for exercising and perceptions of attractiveness in female first year university students. This population is vulnerable to the effects of media and in danger of becoming less active. Therefore, the influence of popular fitness and health magazines on exercise motivation is an important area of research given the popularity of these magazines. The good news is that health messages may be influential and result in arguing against the idea of a “perfect body”. Further, the results provide evidence that health and appearance are not necessarily distinct concepts for female undergraduate students. It was also of interest that perceptions of one’s attractiveness did not differ by exercise identity. Promoting physical activity in a way that will appeal to university students of any weight and exercise status is a challenge in the face of overwhelming conflicting media messages about exercise that exist [18]. However, those who are interested in promoting physical activity and exercise to female university students may wish to highlight that health is more attractive than being overly thin and unfit as this message seems to resonate with this group. This research also provides a starting point for future research examining how ideas of health, exercise, and appearance are influenced by media representations of exercisers and subsequently considered by consumers.
